# Dielectric metasurfaces based on a phase singularity in the region of high reflectance

**DOI:** 10.1515/nanoph-2024-0700

**Published:** 2025-03-18

**Authors:** Jaewon Jang, Minsu Park, Hyeonjeong Kang, Gyu-Won Han, Hui Jae Cho, Yeonsang Park

**Affiliations:** Departement of Physics, Chungnam National University, Daejeon, Korea; Institute of Quantum Systems, Chungnam National University, Deajeon, Korea; Office of Nano Convergence Technology, National NanoFab Center, Deajeon, Korea

**Keywords:** dielectric metasurfaces, cross-shaped structure, high reflectance, phase singularity

## Abstract

Metasurfaces, two-dimensional planar optical devices based on subwavelength-scale structures, have garnered significant attention for their potential to replace conventional optical components in various fields. These devices can manipulate the amplitude, phase, and polarization of light in versatile ways, offering complex functionalities within a single, space-efficient device. However, enhancing their functionality remains a challenge, requiring an expansion in the design flexibility of the structural elements, known as meta-atoms. In this study, we revealed that by varying the two independent lengths of the cross-shaped structure at a wavelength of 980 nm, a phase singularity exists in the region of high reflection. In addition, we found that the phase of transmitted light can be modulated from 0 to 2*π* by encircling this singularity. Based on the identified phase singularity, we designed and fabricated a polarization-independent metalens with varying numerical apertures to experimentally validate the feasibility of high-reflectivity transmissive wavefront engineering metasurfaces. The introduced meta-atoms based on a phase singularity are expected to open new avenues for applications, such as those requiring light attenuation and concentration simultaneously or the development of resonant cavity structures capable of beam modulation.

## 1 Introduction

Implementing optical elements in lighter and more compact forms is a critical challenge that must be addressed to make advanced optical devices space-efficient as they become increasingly complex and bulky. One of the most promising solutions to this problem is metasurfaces, which are flat optical devices based on subwavelength structures. Metasurfaces can flexibly control key characteristics of light, such as amplitude, phase, and polarization, within a single device. Thanks to this potential, various metasurface-based meta-devices have been continuously researched and developed. Recent advancements in metasurfaces have demonstrated their potential to realize groundbreaking devices, such as diffraction-limited focusing lenses [1], transmissive lenses operating in the EUV range [2], full-colour 3D holographic augmented reality (AR) displays [3], and devices for generating vectorial optical fields [4]. Moreover, metasurfaces have been reported to enable mass production at the chip scale through the use of CMOS platforms [5], [6] and nanoimprinting technologies [7], [8], offering a promising solution to overcome the limitations of bulky and heavy conventional optical components. The versatility and potential of metasurfaces have drawn significant attention across various advanced industries, including LiDAR [9], [10], smartphones [11], [12], image sensors [13], [14], biosensing [15], [16], and AR and VR applications [17], [18].

However, for metasurfaces to fully replace conventional optical components and, furthermore, to realize an advanced, independent optical platform based on metasurfaces, there are still many challenges to overcome. These include achieving ultra-broadband coverage, developing methods for fabricating three-dimensional structures at shorter wavelengths, implementing fully tunable metasurfaces, and developing various metasurface-integrated devices. To overcome these challenges, it is essential to develop advanced fabrication technologies and identify methods that can further enrich and enhance the functionality of metasurfaces. Recent research has focused on improving material properties, such as the development of low-loss and high refractive index materials [19], [20], [21], as well as exploring tuning methods based on multiple platforms [22], [23], [24]. Additionally, efforts to expand the structural degrees of freedom have been ongoing, including the design of meta-atoms with diverse geometric structures [25], [26], the implementation of multilayered metasurfaces [27], [28], and the realization of three-dimensional structures [29], [30]. These efforts are expected to overcome the current limitations of metasurfaces and provide a crucial pathway for realizing more complex and multifunctional meta-devices.

Particularly, efforts to expand the structural degrees of freedom are closely linked to advancements in meta-atom design principles. Various methods have been proposed for designing the meta-atoms that make up metasurfaces. Notably, resonant phase [31], [32], propagation phase [33], [34], and geometric Pancharatnam–Berry (PB) phase [35], [36] are representative, and these methods can be combined in various ways to enable a single metasurface to simultaneously achieve multiple functions [37], [38]. In addition, recent approaches have introduced new phase modulation techniques, such as chirality-assisted phase [39] and exceptional-topological phase [40]. Moreover, the rapid advancement of artificial intelligence (AI)-driven non-intuitive design methods demonstrates the vast potential for further expanding the development of metasurfaces [41], [42]. As a result of these efforts, metasurfaces have consistently demonstrated their applicability across various fields, and more recently, they have been actively studied in the field of quantum optics [43], [44], [45].

In this paper, we propose a novel meta-atom design that enables a metasurface to achieve a high reflectance, while simultaneously providing a full 0 to 2*π* phase gradient for the transmitted light. This approach contrasts with traditional transmissive metasurfaces, which typically focus on full phase control and near-unity transmission. Based on this design, we demonstrate the realization of a highly reflective meta-reflector and a metasurface for wavefront engineering of the transmitted light. We found through simulations that when *y*-polarized light in the near-infrared spectrum near 980 nm propagates through a cross-shaped structure made of hydrogenated amorphous silicon (a-Si:H) on a quartz substrate, three magnetic dipole (MD)-like modes are excited along the *x*-axis within the cross-shaped structure, resulting in a pronounced reflective effect. This region with significantly high reflectance exhibits largely in the space defined by two independent length parameters of the cross-shaped structure. Within this region, we could identify a phase singularity that enables the generation of a full 0 to 2*π* phase gradient for the transmitted light, not the reflected light by encircling this point. Based on this finding, we designed a polarization-independent metalens capable of high reflectance while simultaneously enabling wavefront modulation of the residual transmitted light. This was achieved by combining the 12 unit cells surrounding the phase singularity within the parameter space of the isotropic cross-shaped structure. Additionally, we designed a meta-reflector based on the periodic repetition of a single cross-shaped structure. Both metasurfaces are fabricated with a size of 2 mm and experimentally validated in terms of their reflective performance and beam modulation capabilities.

The new degrees of freedom for generating full-phase gradients based on the phase singularity presented here are expected to be applicable not only to the metalens but also to the design of various low-transmission wavefront-modulating meta-devices. In particular, we anticipate that these findings could be applied to resonant structures, such as vertical cavities, that enable beam modulation. Additionally, we believe that the phase singularity existing within the high-reflectance region of this parameter space may be related to fascinating topics in physics, such as orbital angular momentum and optical vortices.

## 2 Phase singularity in the high-reflectance region

**Figure 1:**
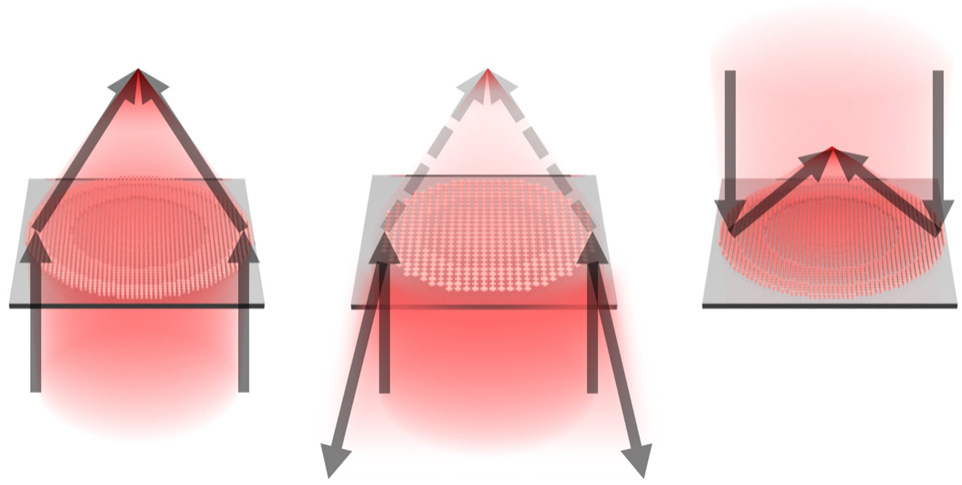
Schematic diagram comparing three distinct types of metasurfaces. (left) Transmissive metalens with high transmittance. (center) Transmissive metalens with high reflectance proposed in this study. (right) Reflective metalens with high reflectance.

Figure 1 presents a comparative schematic that depicts the concept of the metasurface introduced in this study, in contrast to conventional approaches. The left of Figure 1 shows a representative example of a high-transmission metalens, which modulates the transmitted wavefront, while the right of Figure 1 depicts a high-reflectance metalens, which modulates the reflected wavefront. The center of Figure 1 illustrates the metasurface proposed in this work, which simultaneously modulates the transmitted wavefront while achieving high reflectance.

We first investigated prior studies related to meta-reflectors with high reflectance based on various structural designs to design a metasurface that can reflect most of the incident light while simultaneously controlling the phase of the residual transmitted light, as shown in the center of Figure 1. Several meta-reflectors with different functionalities, based on metals or dielectrics, have been proposed to date. Representative examples include meta-mirrors [46], metasurfaces capable of modulating the phase of reflected waves [47], [48], retroreflector [49], and reflective polarization-dependent metasurface holograms [50], [51]. However, these previous studies primarily focused on increasing reflection efficiency and controlling the phase and polarization characteristics of reflected light, which is indeed a reasonable design approach for the development of reflective metasurfaces.

On the other hand, our objective here is to design a metasurface that can modulate the phase of transmitted light, rather than reflected light, while maintaining high reflectance, which may appear somewhat contradictory. To establish the essential meta-atom library for realizing this type of metasurface, we first selected a quartz substrate and a-Si:H, which has a high refractive index, as the platform, ensuring the absence of absorption in the target wavelength range near 980 nm. Next, we focused on identifying meta-atom structures capable of exhibiting high reflectance. As a result, through finite-difference time-domain (FDTD) simulations (Lumerical Inc.), we confirmed that isotropic structures, such as cylinders and square pillars, arranged periodically in a square lattice with a period of 770 nm and a thickness of 210 nm, exhibit very high reflectance in the vicinity of 980 nm (see Supplementary Information S1).

**Figure 2:**
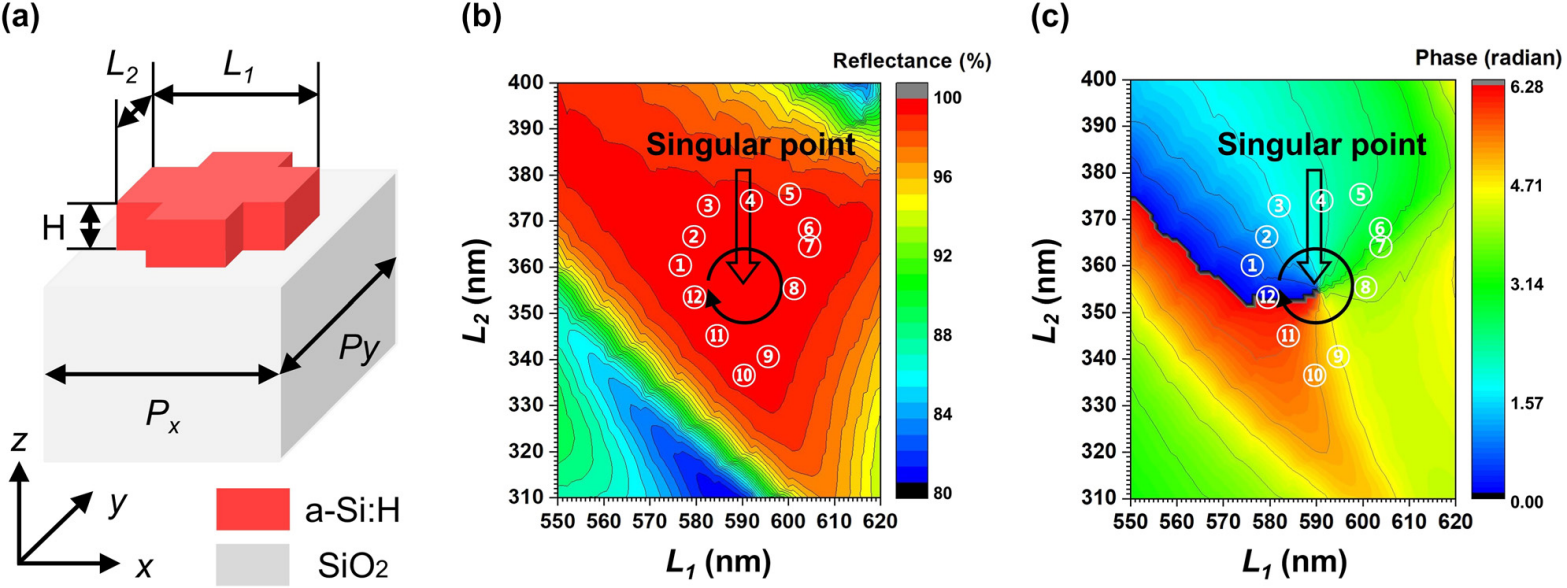
Simulation results for the (*L*_1_, *L*_2_) parameter space of the cross-shaped meta-atom at a wavelength of 980 nm. *L*_1_ and *L*_2_ were calculated with a 1 nm interval. (a) Schematic of the a-Si:H cross-shaped meta-atom unit cell. The meta-atom is arranged in a square lattice with a period of *P*_*x*_ = *P*_*y*_ = 770 nm, where the height is *H* = 210 nm. The long side is denoted as *L*_1_ and the short side as *L*_2_. The substrate corresponds to SiO_2_. (b) Reflectance map calculated over the (*L*_1_, *L*_2_) parameter space, expressed as a percentage. (c) Phase map of the transmitted light calculated over the (*L*_1_, *L*_2_) parameter space, expressed in radians. The location of the singularity is at (591 nm, 355 nm). The 12 unit cells surrounding the singularity, marked by numbers in the figure, correspond to the unit cells used in the metalens design.

To further explore methods for obtaining additional degrees of freedom for modulating the transmitted phase, we adopted a cross-shaped nanostructure with an additional adjustable parameter compared to the previously investigated isotropic structures, as the unit cell, as shown in Figure 2(a). This structure not only exhibits high reflectance but also enables modulation of the transmitted phase. The two length parameters, *L*_1_ and *L*_2_, were treated as variables, and both reflectance and phase were calculated. As a result, the (*L*_1_, *L*_2_) parameter space for the cross-shaped structure still contains regions of high reflectance, as shown in Figure 2(b), while simultaneously revealing a phase singularity that generates a full phase gradient, as depicted in Figure 2(c).

**Figure 3:**
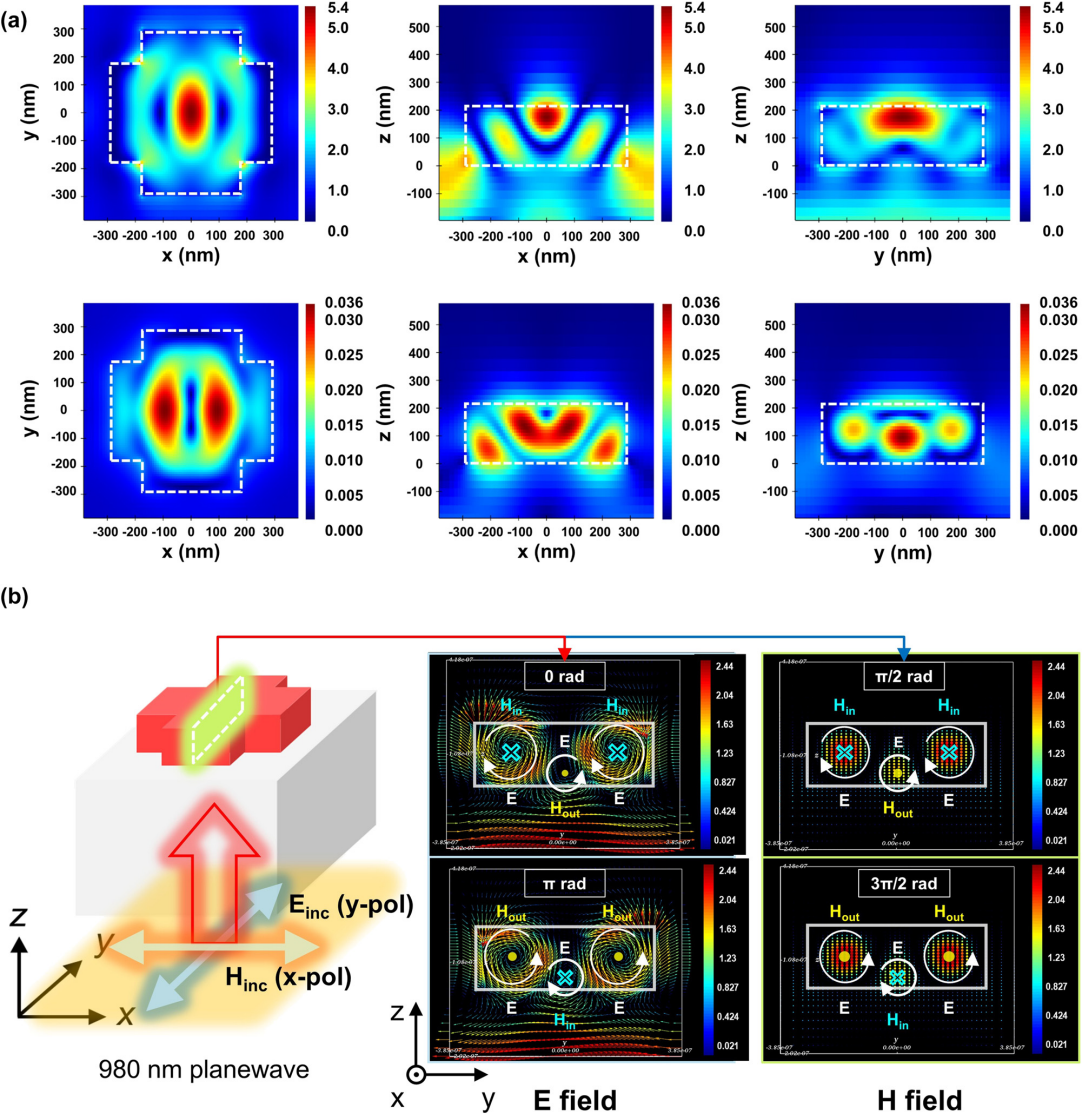
Simulation results for a periodically repeated cross-shaped structure arbitrarily selected in the high reflectance region, with *y*-polarized light at a wavelength of 980 nm incident from below. (a) Electromagnetic field profile formed in the unit cell. The top and bottom represent the electric and magnetic field profiles, respectively, and the *xy* cross-sectional profiles are extracted at *z* = 170 nm. Each field is represented as an absolute value. (b) Electromagnetic vector field profile formed in the unit cell.

**Figure 4:**
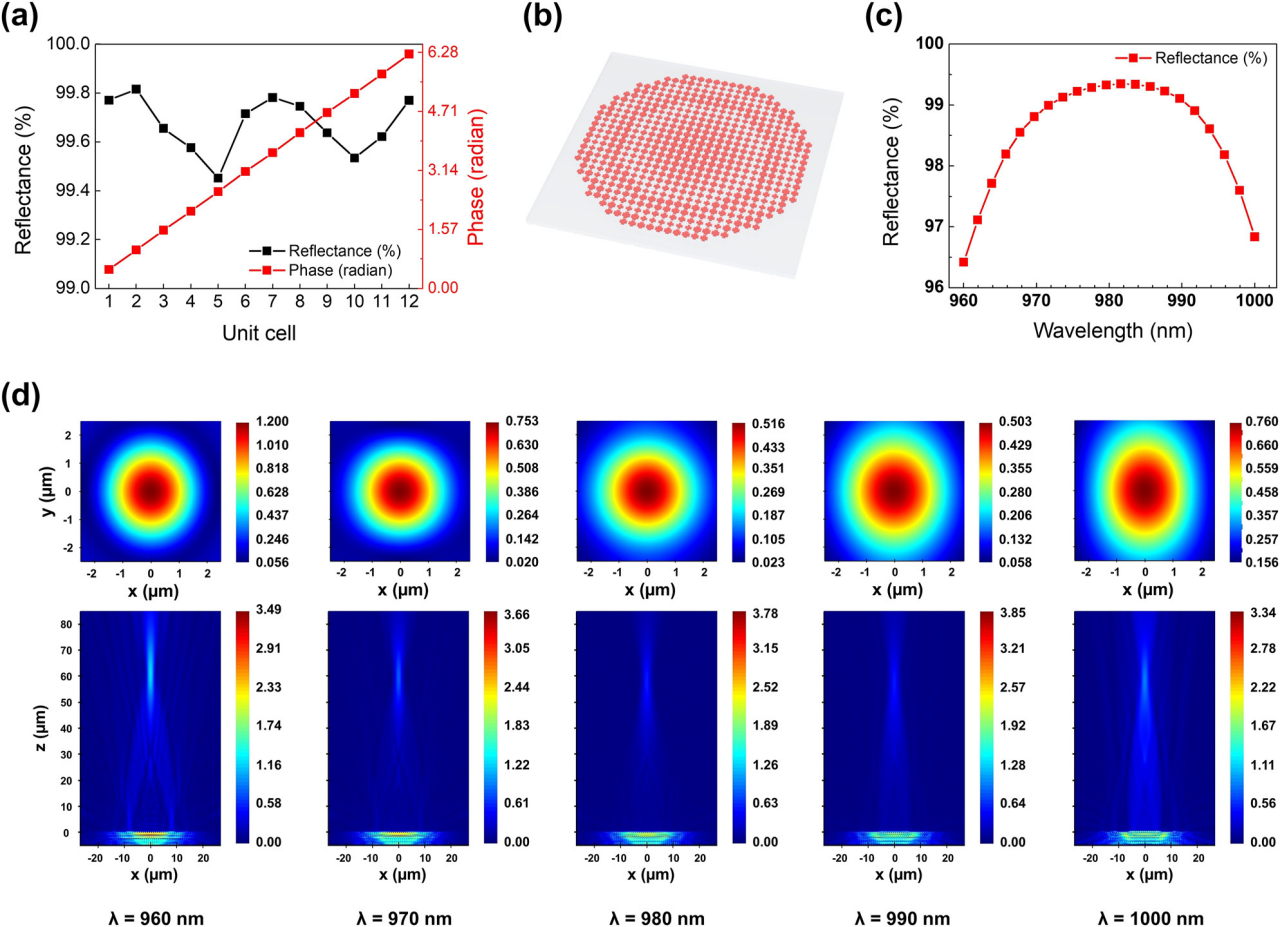
Design and simulation results of the phase singularity-based metalens. (a) Reflectance and phase calculated for the 12 unit cells that constitute the metalens. (b) Schematic of the cross-shaped structure-based metalens. (c) Reflectance calculated for the designed metalens over the wavelength range from 960 nm to 1,000 nm, with a 2 nm wavelength interval. (d) Simulation results of high-reflected and focused transmitted light. Field profiles are absolute values of electric fields in the *xy* (top) and *xz* (bottom) cross-sections calculated at each wavelength shown in bottom textboxes. Top *xy* cross-sections were extracted at *z* = 61.96 µm, which corresponds to the sum of the focal length (61.75 µm) and the metalens height (210 nm).

To investigate the origin of the high reflectance mechanism and the generation of a phase singularity that can produce a full phase gradient in the (*L*_1_, *L*_2_) parameter space of these dielectric high refractive index cross-shaped meta-atom structures, we performed detailed electromagnetic (EM) simulations and adapted a multi-mode decomposition analysis [52], [53], [54]. As a result, when *y*-polarized light with a wavelength of 980 nm is incident from a quartz onto the a-Si:H cross-shaped structure, a partially localized EM field is formed within the structure as shown in Figure 3(a). From field simulation results shown in Figure 3(a), we can see that electric dipole-like (ED-like) and magnetic dipole-like (MD-like) mode are constructed in the suggested structure and changing each other continuously. This phenomenon can be clearly seen by visualizing electromagnetic (EM) fields as vector forms as shown in Figure 3(b). From the electric field vector profile shown in the top-left position of Figure 3(b), we can see that the electric field vectors at the top of the structure form the right-handed loops and those at the bottom of the structure form the left-handed loop. From the vector profile shown in the bottom-left position of Figure 3(b), it is shown that the direction of field loop was changed oppositely, after the phase of *π* radian (half-period time) is changed. Similarly, we can also see the same phenomenon in the magnetic field shown in the right-column of Figure 3(b) clearly. This shows that ED-like and MD-like modes are changing their direction periodically and resultantly makes direction scattering with high reflection. To make sure of high reflection, we also checked out the reflection value at each wavelength as shown in Figure 4(c) and visualized them as video movie. (Additional EM analysis data can be found in Supplementary Information S2 and see Supplementary Movies).

Additionally, it has been well established in previous studies that Mie resonance modes formed inside high refractive index dielectric structures, such as spherical or cylindrical shapes, are capable of reflecting incident light or scattering it directionally [55], [56], [57], [58]. Based on these prior findings, it can be anticipated that the cross-shaped structure presented here also exhibits high reflectance due to the Mie resonance effect. However, the modes formed in the proposed cross-shaped structure do not correspond to the single MD or electric dipole (ED) modes typically observed in previous studies, but rather appear to exhibit more complex multi-mode behaviour. Furthermore, to demonstrate that the observed high reflectance is not solely due to the unique shape of the cross-shaped structure, but rather results from the specific Mie modes commonly formed in high refractive index nanostructures with similar aspect ratios, we performed identical EM-field calculations and comparative analyses on isotropic structures of similar size (see Supplementary Information S1). As a result, it was confirmed that similar multiple ED-like and MD-like modes are formed in isotropic structures such as cylinders and square pillars at comparable positions to those in the cross-shaped structure. This suggests that the high reflectance is attributed to the common Mie resonance modes.

To the best of our knowledge, the origin of the phase singularity existing in the two-dimensional length parameter space of the cross-shaped structure has not yet been elucidated. In this study, although we did not establish a clear correlation regarding the origin of the phase singularity arising from the adjustment of the length parameters of the cross-shaped structure, we speculate that the degrees of variation in the individual ED-like and MD-like modes formed inside the structure, as *L*_1_ and *L*_2_ change independently, influence the transmitted phase. Indeed, as shown in Figure 2(b) and (c), when the structure is modified in the direction of decreased reflectance, the phase gradient nearly disappears, and the initially identified Mie resonance modes are gradually deformed (see Supplementary Information S2). We anticipate that the development of a precise multi-mode decomposition analysis method for the periodic array of these dielectric cross-shaped structures will allow for the individual decomposition of the Mie resonance modes formed within these complex arrays, providing dominance information of the most significant modes. We expect that this will offer valuable insights not only into the reflection mechanism but also into the underlying principles governing the formation of phase singularity.

## 3 Metalens based on a phase singularity

We selected 12 meta-atoms around the phase singularity as unit cells denoted by numbers in Figure 2(b) and (c), and used them to design a metalens, a representative wavefront engineering meta-device. By fabricating and measuring the metalens, we demonstrated the phase modulation capabilities of the cross-shaped structure meta-atoms. Figure 4(a) shows the reflectance and transmitted phase values at a wavelength of 980 nm for the 12 selected unit cells. It is observed that all selected unit cells exhibit a reflectance of over 99.45 % and can implement a full phase gradient from 0 to 2*π*. Table 1 provides the numerical values of reflectance and transmitted phase along with the corresponding (*L*_1_, *L*_2_) values for each of the selected 12 unit cells. Figure 4(b) illustrates a schematic of the designed metalens based on the selected unit cells, with a diameter of 53.9 µm, numerical aperture (NA) of 0.4, and a focal length of 61.75 µm. This metalens can be designed using eq. (1), which represents the phase distribution of the lens. The relationship between the NA and focal length (*f*) of the metalens is defined by eq. (2). *r* in eq. (1) and *R* in eq. (2) correspond to the radial position of each element and the radius of the designed metalens, respectively. In eq. (1), *λ* represents the design wavelength. Due to limitations in computational resources, calculations were not performed for the exact specifications corresponding to the actual device with a 2 mm diameter. However, since both the simulations and the fabricated samples were based on the same design principles, consistency between the design and fabrication processes is maintained. The calculated reflectance of the designed metalens is shown in Figure 4(c). The metalens exhibits a reflectance of approximately 99.33 % at 980 nm and maintains a high reflectance of over 96.42 % across a 40 nm bandwidth, ranging from 960 nm to 1,000 nm. It is observed that the metalens shows a slightly lower reflectance compared to the case where a single unit cell of the same size is periodically repeated. This is due to the arrangement of unit cells of different sizes in space, following the phase distribution in eq. (1), which alters the degree of EM coupling between adjacent unit cells [59], [60]. Such effects are expected to be addressed through various design optimization processes [61], [62].

(1) $$\varphi \left(r,\lambda ,f\right)=-\frac{2\pi }{\lambda }\cdot \left(\sqrt{r^{2}+f^{2}}-f\right)\;\text{where}\;r=\sqrt{x^{2}+y^{2}}$$

(2) $$\begin{array}{ll} f_{NA} & =R\cdot \tan\left(\frac{\pi }{2}-\sin^{-1}\left(\text{NA}\right)\right)\\ & \;\text{where}\;R\;\text{is}\;\text{the}\;\text{radius}\;\text{of}\;\text{a}\;\text{metalens}. \end{array}$$

**Table 1:** The numerical values of reflectance and transmitted phase along with the corresponding (*L*_1_, *L*_2_) values for each of the selected 12 unit cells.

Unit cell	1	2	3	4	5	6	7	8	9	10	11	12
*L*_1_ (nm)	577	580	583	592	601	605	605	602	596	591	585	580
*L*_2_ (nm)	359	365	372	373	375	367	364	354	339	335	344	352
*φ*_trans_ (radian)	0.505834	1.02811	1.55177	2.05363	2.57811	3.1142	3.61221	4.14878	4.6808	5.19027	5.70549	6.23885
Reflectance (%)	99.771	99.816	99.655	99.576	99.452	99.716	99.782	99.746	99.637	99.534	99.622	99.770

Furthermore, to evaluate the focusing capability of the designed metalens, we calculated the electric field profiles in both the *xz* and *xy* cross-sections. As shown in Figure 4(d), the metalens effectively forms a focal spot within a 40 nm bandwidth centered at the design wavelength of 980 nm. These results demonstrate that the proposed phase singularity-based metalens can maintain reliable focusing performance, thereby validating the robustness of the design.

## 4 Results and discussion

**Figure 5:**
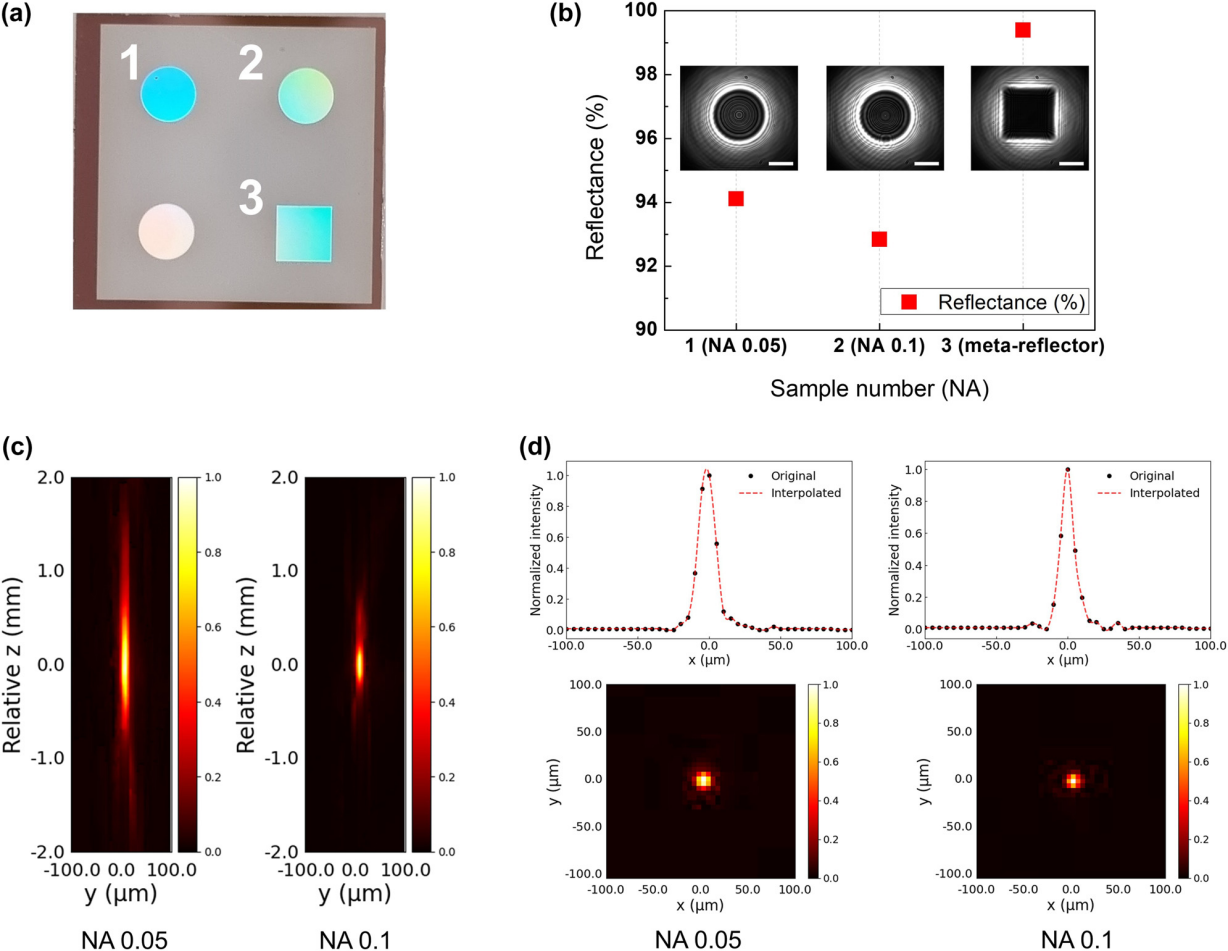
Measured reflectance and beam profiles for the fabricated metasurfaces. (a) Photographs of the metasurface samples. Sample 1, 2 and 3 correspond to the NA 0.05 metalens, the NA 0.1 metalens, and the meta-reflector, respectively. (b) Measured reflectance for the meta-reflector, NA 0.05, and NA 0.1 metalenses using a photodiode, with an inset showing an image captured by the short wavelength infrared wavelength camera. (c) Measured intensity profiles along the optical axis for the NA 0.05 and NA 0.1 metalenses, shown in the *yz* plane. (d) Intensity profiles measured at the focal plane for the NA 0.05 and NA 0.1 metalenses, along with intensity line plot along the *x*-axis.

Here, we select an arbitrary structure with dimensions (*L*_1_, *L*_2_) = (604 nm, 346 nm) from the high-reflectance region within the parameter space. By repeating this structure, we design and fabricate a meta-reflector of size 2 mm by 2 mm (Sample 3 in Figure 5(a)) and two types of metalenses with a diameter of 2 mm and NA values of 0.05 and 0.1 (Samples 1 and 2 in Figure 5(a)). The metalenses are designed using a combination of 12 unit cells, as presented in Table 1. We then present and analyze the experimental results for each of these metasurfaces. The former was fabricated for the purpose of verifying the high reflectance of the proposed cross-shaped structure-based metasurface, while the latter was designed to simultaneously validate both the high reflectance and the ability to modulate the transmitted wavefront. A single-mode pump laser with a wavelength of 974.5 nm was used as the light source (Detailed experimental setup used in the experiments can be found in Supplementary Information S3). The experimental results show that the fabricated meta-reflector achieves a high reflectance of 99.39 %, while the two metalenses, fabricated with NA values of 0.05 and 0.1, respectively, exhibit reflectance values of 94.10 % and 92.83 %, and can form a focal spot.

### 4.1 High reflectance of metasurfaces

First, to experimentally verify the reflective performance of the proposed cross-shaped structure-based metasurface, reflectance measurements were performed on the meta-reflector and two metalenses with NA values of 0.05 and 0.1, respectively. A 974.5 nm laser was incident on the samples, and the intensity of the transmitted light was measured using a photodiode sensor. The graph in Figure 5(b) shows the reflectance values measured for the meta-reflector and the NA 0.05 and NA 0.1 metalenses, while the inset image captures the transmitted laser beam passing through each sample, taken with a short wavelength infrared (SWIR) camera. The meta-reflector, which is composed of repeating single structures, exhibits a higher reflectance compared to the metalenses, which are composed of different unit cell combinations. This difference is likely due to variations in the sizes of adjacent structures in the design of the metalenses, leading to changes in the electromagnetic interaction between the structures. Additionally, the discrepancies observed between the theoretical and experimental results for both the meta-reflector and the metalenses are likely attributed to dimensional errors in the fabricated cross-shaped structures, such as blunt corner shapes, as well as pattern dimension errors arising from the photolithography and etching processes. These effects are considered the primary contributors to the reduction in reflectance. (A detailed analysis of the error factors can be found in Supplementary Information S4.)

### 4.2 Wavefront engineering of metasurfaces

Next, we conducted beam imaging experiments to verify the wavefront modulation capabilities of the fabricated metalenses. The measured metalenses, labeled as Sample 1 and Sample 2 in Figure 5(a), were designed and fabricated with diameters of 2 mm and NA of 0.05 and 0.1, respectively. Figure 5(c) shows the intensity profiles measured along the optical axis for each of the metalenses. The images were collected within a ±2 mm range around the focal plane (*z* = 0 mm) formed by each of the metalenses, and both metalenses exhibited consistent focusing capabilities. Notably, the metalens with a lower NA (0.05) demonstrated a significantly longer depth of focus compared to the NA 0.1 metalens. Figure 5(d) shows the intensity profiles at the focal plane, along with the intensity line plot along the *x*-axis. The black points represent the intensity measured at the pixel level, while the red dashed lines correspond to the interpolated data. This interpolation was used to compensate for the limitation of the SWIR camera used in this experiment, which has a minimum pixel size of 5 µm by 5 µm, making it difficult to precisely measure the beam size with higher resolution. From the interpolation results, we observe that the focal spot full width at half maximum (FWHM) values formed by the NA 0.05 and NA 0.1 metalenses are approximately 13.814 µm and 10.611 µm, respectively.

## 5 Conclusions

In this study, we propose a metasurface that can modulate the transmitted wavefront while maintaining high reflection, moving away from the conventional design approaches of most existing meta-devices, which primarily emphasize high transmittance and high reflectance. To achieve this, we explored structures with high reflectivity and a high degree of freedom for phase modulation of the transmitted wave, using non-absorptive a-Si:H and a quartz substrate at the target wavelength near 980 nm. As a result, we identified a region of very high reflectivity within the parameter space of the two length variables of the cross-shaped structure, along with a phase singularity that exhibits a full phase gradient. Based on this, we designed a meta-reflector with extremely high reflectivity and transmissive metalenses that maintains high reflectivity while allowing phase modulation of the remaining small amount of transmitted light. Furthermore, based on the proposed design, we fabricated meta-reflectors and meta-lenses with NA 0.05 and 0.1 at millimeter scale, and experimentally demonstrated the feasibility of achieving high reflectivity and transmitted beam modulation through optical characterization. We believe that this metasurface design approach can still be realized not only at a wavelength of 980 nm but also across a wide range of wavelengths by using high refractive index dielectrics. These metasurfaces are expected to be a promising option for applications such as cavity structures with beam modulation and low-transmittance beam control (see Supplementary Information S5). Finally, we believe that the phase distribution around this phase singularity, which appears to be related to optical vortices and seems to possess orbital angular momentum, may carry more intriguing physical implications.

## Supplementary Material

Supplementary Material Details

## References

[1] Khorasaninejad M., Chen W. T., Devlin R. C., Oh J., Zhu A. Y., Capasso F. (2016). Metalenses at visible wavelengths: diffraction-limited focusing and subwavelength resolution imaging. *Science*.

[2] Ossiander M. (2023). Extreme ultraviolet metalens by vacuum guiding. *Science*.

[3] Gopakumar M. (2024). Full-colour 3D holographic augmented-reality displays with metasurface waveguides. *Nature*.

[4] Wang D., Liu F., Liu T., Sun S., He Q., Zhou L. (2021). Efficient generation of complex vectorial optical fields with metasurfaces. *Light: Sci. Appl.*.

[5] Li N. (2020). Large-area metasurface on CMOS-compatible fabrication platform: driving flat optics from lab to fab. *Nanophotonics*.

[6] Park J.-S. (2024). All-glass 100 mm diameter visible metalens for imaging the cosmos. *ACS Nano*.

[7] Kim J. (2023). One-step printable platform for high-efficiency metasurfaces down to the deep-ultraviolet region. *Light: Sci. Appl.*.

[8] Ko B. (2022). Tunable metasurfaces via the humidity responsive swelling of single-step imprinted polyvinyl alcohol nanostructures. *Nat. Commun.*.

[9] Kim I. (2021). Nanophotonics for light detection and ranging technology. *Nat. Nanotechnol.*.

[10] Juliano Martins R. (2022). Metasurface-enhanced light detection and ranging technology. *Nat. Commun.*.

[11] Kim Y. (2024). Metasurface folded lens system for ultrathin cameras. *Sci. Adv.*.

[12] Joo W.-J. (2020). Metasurface-driven OLED displays beyond 10,000 pixels per inch. *Science*.

[13] Kim C. (2024). Freeform metasurface color router for deep submicron pixel image sensors. *Sci. Adv.*.

[14] Lee J. (2022). Compact meta-spectral image sensor for mobile applications. *Nanophotonics*.

[15] Zhang S. (2020). Metasurfaces for biomedical applications: imaging and sensing from a nanophotonics perspective. *Nanophotonics*.

[16] Tseng M. L., Jahani Y., Leitis A., Altug H. (2020). Dielectric metasurfaces enabling advanced optical biosensors. *ACS Photonics*.

[17] Lee G.-Y. (2018). Metasurface eyepiece for augmented reality. *Nat. Commun.*.

[18] Li Z., Pestourie R., Park J.-S., Huang Y.-W., Johnson S. G., Capasso F. (2022). Inverse design enables large-scale high-performance meta-optics reshaping virtual reality. *Nat. Commun.*.

[19] Park C.-S., Koirala I., Gao S., Shrestha V. R., Lee S.-S., Choi D.-Y. (2019). Structural color filters based on an all-dielectric metasurface exploiting silicon-rich silicon nitride nanodisks. *Opt. Express*.

[20] Yang Y. (2021). Revealing structural disorder in hydrogenated amorphous silicon for a low-loss photonic platform at visible frequencies. *Adv. Mater.*.

[21] Goldberg O., Mazurski N., Levy U. (2024). Silicon rich nitride: a platform for controllable structural colors. *Nanophotonics*.

[22] Jung C., Lee E., Rho J. (2024). The rise of electrically tunable metasurfaces. *Sci. Adv.*.

[23] Komar A. (2018). Dynamic beam switching by liquid crystal tunable dielectric metasurfaces. *ACS Photonics*.

[24] Kim Y. (2019). Phase modulation with electrically tunable vanadium dioxide phase-change metasurfaces. *Nano Lett.*.

[25] Shi T. (2022). Planar chiral metasurfaces with maximal and tunable chiroptical response driven by bound states in the continuum. *Nat. Commun.*.

[26] Zhou C. (2023). Bound states in the continuum in asymmetric dielectric metasurfaces. *Laser Photonics Rev.*.

[27] Zhou Y., Kravchenko I. I., Wang H., Zheng H., Gu G., Valentine J. (2019). Multifunctional metaoptics based on bilayer metasurfaces. *Light: Sci. Appl.*.

[28] Chang T. (2022). Universal metasurfaces for complete linear control of coherent light transmission. *Adv. Mater.*.

[29] Xu H.-X. (2021). Polarization-insensitive 3D conformal-skin metasurface cloak. *Light: Sci. Appl.*.

[30] Tseng M. L. (2019). Stress-induced 3D chiral fractal metasurface for enhanced and stabilized broadband near-field optical chirality. *Adv. Opt. Mater.*.

[31] Meinzer N., Barnes W. L., Hooper I. R. (2014). Plasmonic meta-atoms and metasurfaces. *Nat. Photonics*.

[32] Wang S. (2017). Broadband achromatic optical metasurface devices. *Nat. Commun.*.

[33] Yu N. (2011). Light propagation with phase discontinuities: generalized laws of reflection and refraction. *Science*.

[34] Khorasaninejad M. (2016). Polarization-insensitive metalenses at visible wavelengths. *Nano Lett.*.

[35] Bomzon Z. E., Biener G., Kleiner V., Hasman E. (2002). Space-variant Pancharatnam–Berry phase optical elements with computer-generated subwavelength gratings. *Opt. Lett.*.

[36] Marrucci L., Manzo C., Paparo D. (2006). Pancharatnam-Berry phase optical elements for wave front shaping in the visible domain: switchable helical mode generation. *Appl. Phys. Lett.*.

[37] Khalid A. U. R., Feng F., Ullah N., Yuan X., Somekh M. G. (2022). Exploitation of geometric and propagation phases for spin-dependent rational-multiple complete phase modulation using dielectric metasurfaces. *Photonics Res.*.

[38] Balthasar Mueller J., Rubin N. A., Devlin R. C., Groever B., Capasso F. (2017). Metasurface polarization optics: independent phase control of arbitrary orthogonal states of polarization. *Phys. Rev. Lett.*.

[39] Yuan Y. (2020). Independent phase modulation for quadruplex polarization channels enabled by chirality-assisted geometric-phase metasurfaces. *Nat. Commun.*.

[40] Song Q., Odeh M., Zúñiga-Pérez J., Kanté B., Genevet P. (2021). Plasmonic topological metasurface by encircling an exceptional point. *Science*.

[41] Liu Z., Zhu D., Rodrigues S. P., Lee K.-T., Cai W. (2018). Generative model for the inverse design of metasurfaces. *Nano Lett.*.

[42] Chen M. K., Liu X., Sun Y., Tsai D. P. (2022). Artificial intelligence in meta-optics. *Chem. Rev.*.

[43] Solntsev A. S., Agarwal G. S., Kivshar Y. S. (2021). Metasurfaces for quantum photonics. *Nat. Photonics*.

[44] Santiago-Cruz T. (2022). Resonant metasurfaces for generating complex quantum states. *Science*.

[45] Zhang D. (2022). All-optical modulation of quantum states by nonlinear metasurface. *Light: Sci. Appl.*.

[46] Cai T. (2021). Ultrawideband chromatic aberration-free meta-mirrors. *Adv. Photonics*.

[47] Ma Z. (2016). Terahertz all-dielectric magnetic mirror metasurfaces. *ACS Photonics*.

[48] Liu B., He Y., Wong S. W., Li Y. (2021). Multifunctional vortex beam generation by a dynamic reflective metasurface. *Adv. Opt. Mater.*.

[49] Arbabi A., Arbabi E., Horie Y., Kamali S. M., Faraon A. (2017). Planar metasurface retroreflector. *Nat. Photonics*.

[50] Zheng G., Mühlenbernd H., Kenney M., Li G., Zentgraf T., Zhang S. (2015). Metasurface holograms reaching 80% efficiency. *Nat. Nanotechnol.*.

[51] Wen D. (2015). Helicity multiplexed broadband metasurface holograms. *Nat. Commun.*.

[52] Wang M. (2020). Suppressing material loss in the visible and near-infrared range for functional nanophotonics using bandgap engineering. *Nat. Commun.*.

[53] Fang J. (2021). Directional modulation of exciton emission using single dielectric nanospheres. *Adv. Mater.*.

[54] Yao K. (2024). Tuning multipolar Mie scattering of particles on a dielectric-covered mirror. *ACS Nano*.

[55] Huang Y., Xu H., Lu Y., Chen Y. (2018). All-dielectric metasurface for achieving perfect reflection at visible wavelengths. *J. Phys. Chem. C*.

[56] Matiushechkina M., Evlyukhin A. B., Zenin V. A., Chichkov B. N., Heurs M. (2024). Perfect mirror effects in metasurfaces of silicon nanodisks at telecom wavelength. *Adv. Opt. Mater.*.

[57] Fu Y. H., Kuznetsov A. I., Miroshnichenko A. E., Yu Y. F., Luk’yanchuk B. (2013). Directional visible light scattering by silicon nanoparticles. *Nat. Commun.*.

[58] Liu T., Xu R., Yu P., Wang Z., Takahara J. (2020). Multipole and multimode engineering in Mie resonance-based metastructures. *Nanophotonics*.

[59] Ollanik A. J., Smith J. A., Belue M. J., Escarra M. D. (2018). High-efficiency all-dielectric Huygens metasurfaces from the ultraviolet to the infrared. *ACS Photonics*.

[60] Zhao W. (2016). Dielectric Huygens’ metasurface for high-efficiency hologram operating in transmission mode. *Sci. Rep.*.

[61] Cai H. (2020). Inverse design of metasurfaces with non-local interactions. *npj Comput. Mater.*.

[62] Gigli C., Li Q., Chavel P., Leo G., Brongersma M. L., Lalanne P. (2021). Fundamental limitations of Huygens’ metasurfaces for optical beam shaping. *Laser Photonics Rev.*.

