# Supplementary material for: Dielectric metasurfaces based on a phase singularity in the region of high reflectance
Source: Nanophotonics. 2025 Mar 18;14(8):1291–300. doi: 10.1515/nanoph-2024-0700 (PMC12019940; doi:10.1515/nanoph-2024-0700)
Supplement: Supplementary file 1 — Supplementary Material Details [file j_nanoph-2024-0700_suppl_001.docx]

**Supplementary Information**

**Dielectric metasurfaces based on a phase singularity in the high-reflectance region**

Jaewon Jang^1,2^, Minsu Park^1,2^, Hyeonjeong Kang^1,2^, Gyu-Won Han^3^, Hui Jae Cho^3^ and Yeonsang Park^1,2^*

*^1^Departement of Physics, Chungnam National University, Daejeon, Korea*

*^2^Instutte of Quantum Systems, Chungnam National University, Deajeon, Korea*

*^3^Office of Nano Convergence Technology, National NanoFab Center, Deajeon, Korea*

** E-mail:* [*yeonsang.park@cnu.ac.kr*](mailto:yeonsang.park@cnu.ac.kr)

**S1. Simulation results and analysis of different isotropic structures based on high refractive index dielectrics**

We demonstrate that the high reflectivity observed in the a-Si:H cross-shaped structures on a quartz substrate results from specific Mie resonant modes formed within the high refractive index dielectric, rather than the geometric shape of the cross itself. By calculating the reflectance spectra for other isotropic structures, such as cylinders and square pillars with similar dimensions, we confirm that these structures also exhibit high-reflectance spectra through finite-difference time-domain (FDTD) simulations. Analysis of the electromagnetic field and vector profiles further reveals that similar Mie resonant modes are formed in all these structures. This indicates that the specific Mie resonant modes in high refractive index dielectrics are responsible for the observed high reflectivity.

Figure S1 shows the calculated reflectance spectra for various isotropic structures near 980 nm, along with the electromagnetic field and vector profiles at this wavelength. In Fig. S1(a), the reflectance spectra for each structure are presented. Simulations were performed under identical conditions, with a square lattice structure having a period of 770 nm and the height of the a-Si:H structures set to 210 nm. The black line represents the reflectance spectrum for the cross-shaped structure at singularity positions ($L_{1}$, $L_{2}$ = 591, 355), the red line for a cylinder with a diameter of 591 nm, and the blue line for a square pillar with a linewidth of 520 nm. Figure S1(b) shows the electromagnetic field and vector profiles at 980 nm for each structure, demonstrating similar field patterns across all configurations.


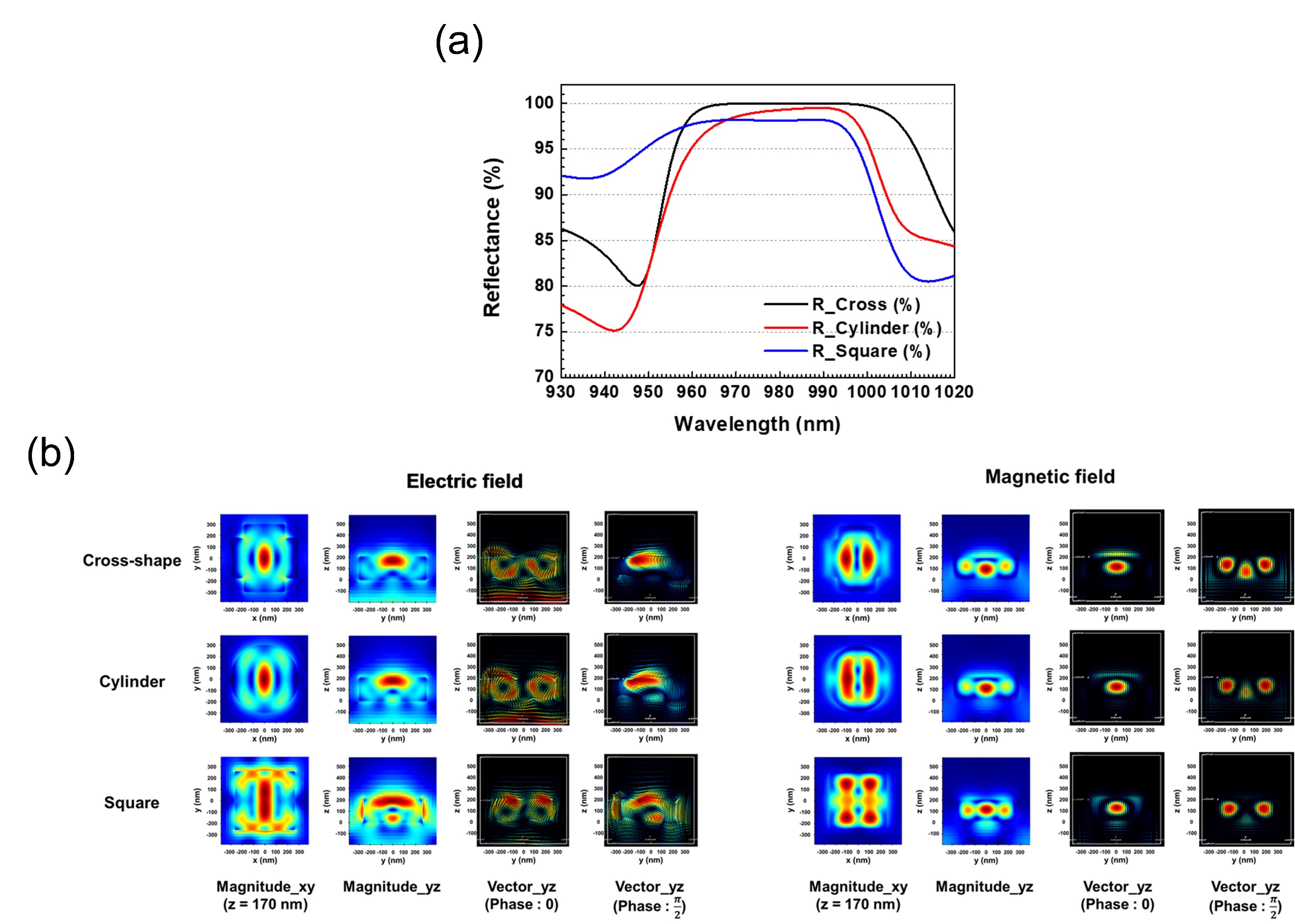


**Figure S1:** Simulation results for different isotropic structures. (a) Reflectance calculated for each of the cross-shaped structure, cylindrical structure, and square pillar structure with a 100 nm bandwidth centered at a wavelength of 980 nm. (b) Electromagnetic field profile and vector profile calculated at 980 nm for each isotropic structure.

**S2. Additional analysis of the electromagnetic field formed in the cross-shaped structure**

Here, we provide a more detailed analysis of the electromagnetic fields formed in the cross-shaped structure. First, we present the component-wise electromagnetic field calculation results for the cross-shaped structure with the same dimensions as shown in Fig. 3(a) of the main text, in Fig. S2(a). The red and blue boxes represent the electric and magnetic field data, respectively. Since this calculation is for y-polarized incident light, as in the main text, we have excluded dummy data.

Additionally, as mentioned in S1, to demonstrate the direct correlation between the specific Mie resonant modes and high reflectance formed in high refractive index media, we conducted a similar analysis of the electromagnetic field profiles for progressively smaller structures, starting from the singularity points in the reflectance and phase maps shown in Fig. 2(b) and (c) of the maintext, and moving toward the bottom left. The selected dimensions were chosen in a sequential manner, starting from the phase singularity point ($L_{1}$, $L_{2}$ = 591, 355) and progressively decreasing in size, with the dimensions being ($L_{1}$, $L_{2}$ = 581, 344), ($L_{1}$, $L_{2}$ = 571, 333), and ($L_{1}$, $L_{2}$ = 561, 322). As shown in the calculation results in Fig. S2(b), we observed that the initially identified Mie resonance modes gradually deformed as the size changed, and, as seen in Fig. 2(b) of the maintext, the reflectance decreased. This indicates that the high reflectance observed in the cross-shaped structure presented in the main text is primarily attributed to the initially identified complex Mie modes.


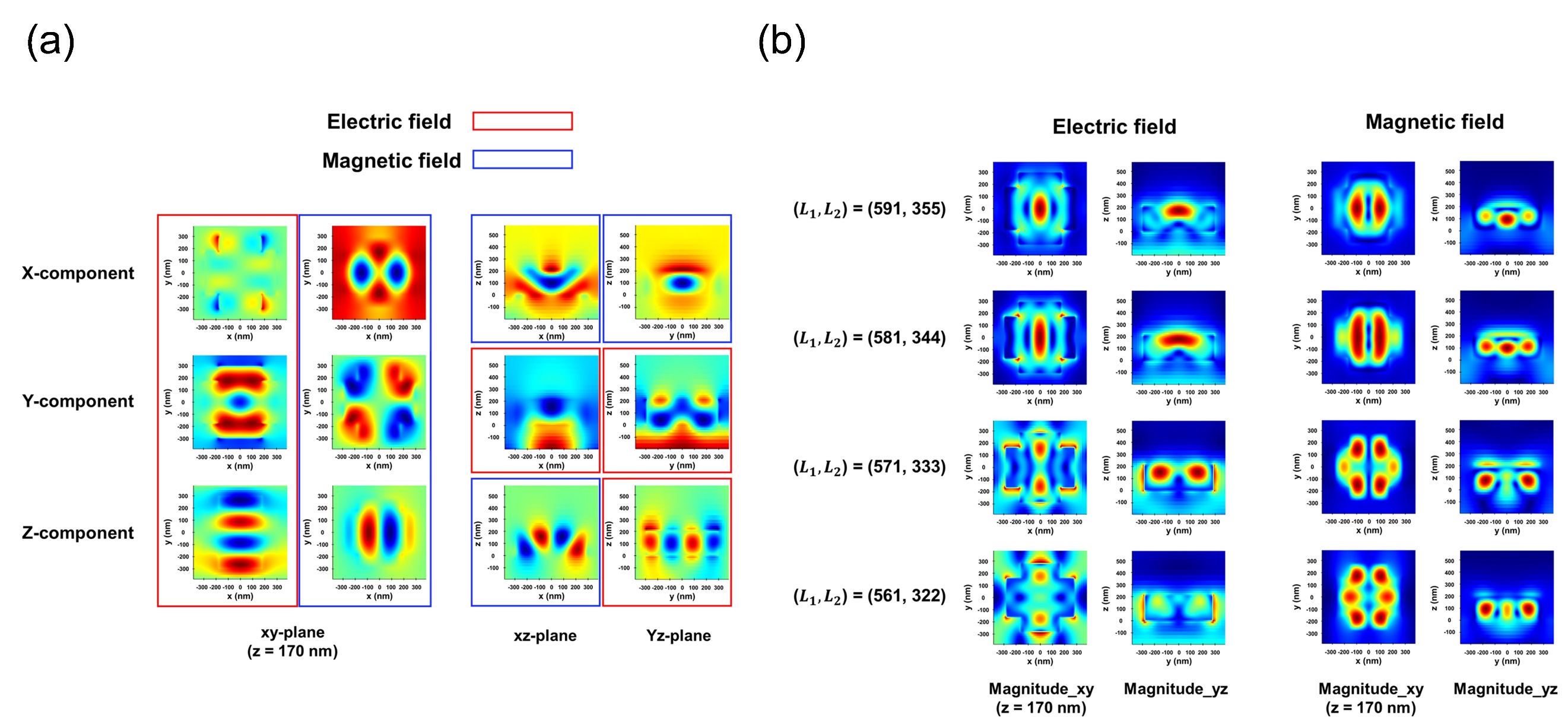


**Figure S2:** Additional simulation results for the cross-shaped structure at 980 nm. (a) Component-wise electromagnetic field profiles for the cross-shaped structure with ($L_{1}$, $L_{2}$ = 591, 355). The red box represents the electric field, and the blue box represents the magnetic field. (b) Electromagnetic field calculation results for the cross-shaped structure as its dimensions decrease in size.

**S3. Experimental setup**

Here, we present the experimental setups used for reflectance measurements and beam imaging experiments. First, Figure S3(a) illustrates the schematic of the experimental setup used for the reflectance measurement of each metasurface. A 974.5 nm single-mode pump laser (1999CHP-3CN01350GL, 3SP Technologies) was used as the light source, driven by a temperature-controlled laser diode driver (CLD1015, Thorlabs Inc.). The laser beam output from the single-mode fiber was collimated into parallel light using a fiber collimator (F280APC-980, Thorlabs Inc.), maintaining a beam diameter of approximately 4 mm. The diameter of the laser beam maintained a level of approximately 4 mm. The laser beam was then incident on a sample of 2 mm in size, and the transmitted light passed through an iris (ID15, Thorlabs) sized to match the sample. The transmitted light was subsequently measured using an InGaAs-based photodiode power sensor (S144C, Thorlabs) and an optical power meter (PM100D, Thorlabs).

Figure S3(b) shows the schematic of the experimental setup used to verify the focusing ability of the two metalenses with numerical apertures (NA) of 0.05 and 0.1. In this configuration, an iris was placed in front of the metalens to sufficiently reduce the distance between the metalens and the CMOS image sensor of the SWIR camera (STC-LBS132POE-SWIR, OMRON SENTECH CO., LTD.). Additionally, to directly collect the focused light passing through the metalens onto the CMOS image sensor, the camera was positioned on a motorized stage. The motorized stage was moved along the optical axis at a speed of 10 µm/s using a motion controller while capturing the images.


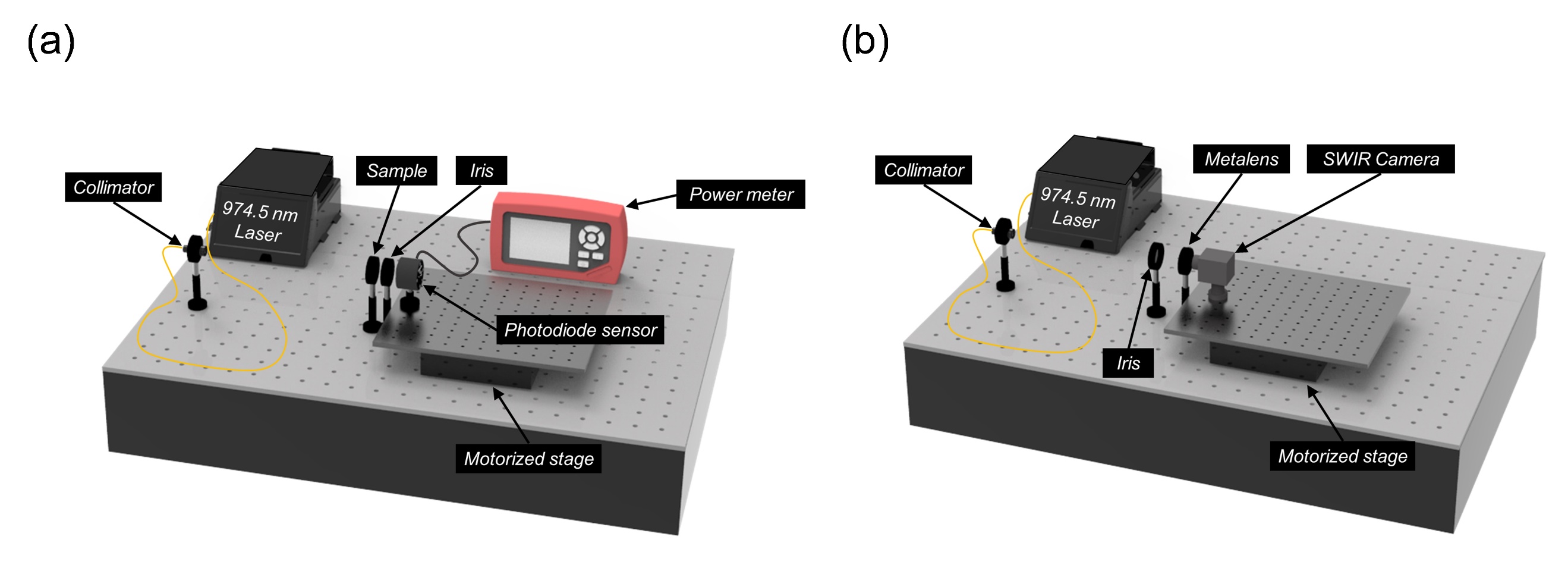


**Figure S3:** Schematic of the experimental setup used for reflectance and beam imaging measurements. (a) Schematic of the reflectance measurement setup. (b) Schematic of the beam imaging measurement setup.

**S4. Analysis of the dominant factors reducing reflectance**

In this section, we analyze the causes of the discrepancy between the measured and calculated reflectance values for the fabricated samples and discuss the dominant factors contributing to the error. We identify two major factors that reduce reflectance and analyze their impact. The first factor is the effect of the blunt corners in the cross-shaped structure, and the second is the dimensional errors in the patterns resulting from the exposure and etching processes. Figure S4(a) shows the SEM images of the three metasurface samples presented in the main text. The left column contains the full images of each sample, while the right column shows magnified images (35,000x) of the central regions of the samples. Sample 1 is a metalens with a diameter of 2 mm designed with NA 0.05, Sample 2 is a metalens with a diameter of 2 mm designed with NA 0.1, and Sample 3 is a 2 mm by 2 mm meta-reflector formed by repeating a structure with ($L_{1}$, $L_{2}$) = (604 nm, 346 nm). As seen in the images on the right column, blunt corner features are formed during the patterning process. To quantitatively analyze the impact of these blunt corner shapes of the cross-shaped structure on reflectance, we examined the corner shape dependence for each of the 12 unit cells used in the metalens design. Figure S4(b) shows the calculated reflectance values for each unit cell with different corner sizes. The results confirm that as the corners of the cross-shaped structure become blunter, the overall reflectance decreases. We anticipate that this issue can be addressed through the incorporation of Optical Proximity Correction (OPC) or by improving the etching recipe.

Moreover, as observed in the reflectance map in Fig.2(b) of the maintext and in Fig. S2(b), the dimensional errors between the actual designed structure and the patterns formed during the patterning process also emerge as significant factors impeding reflectance.


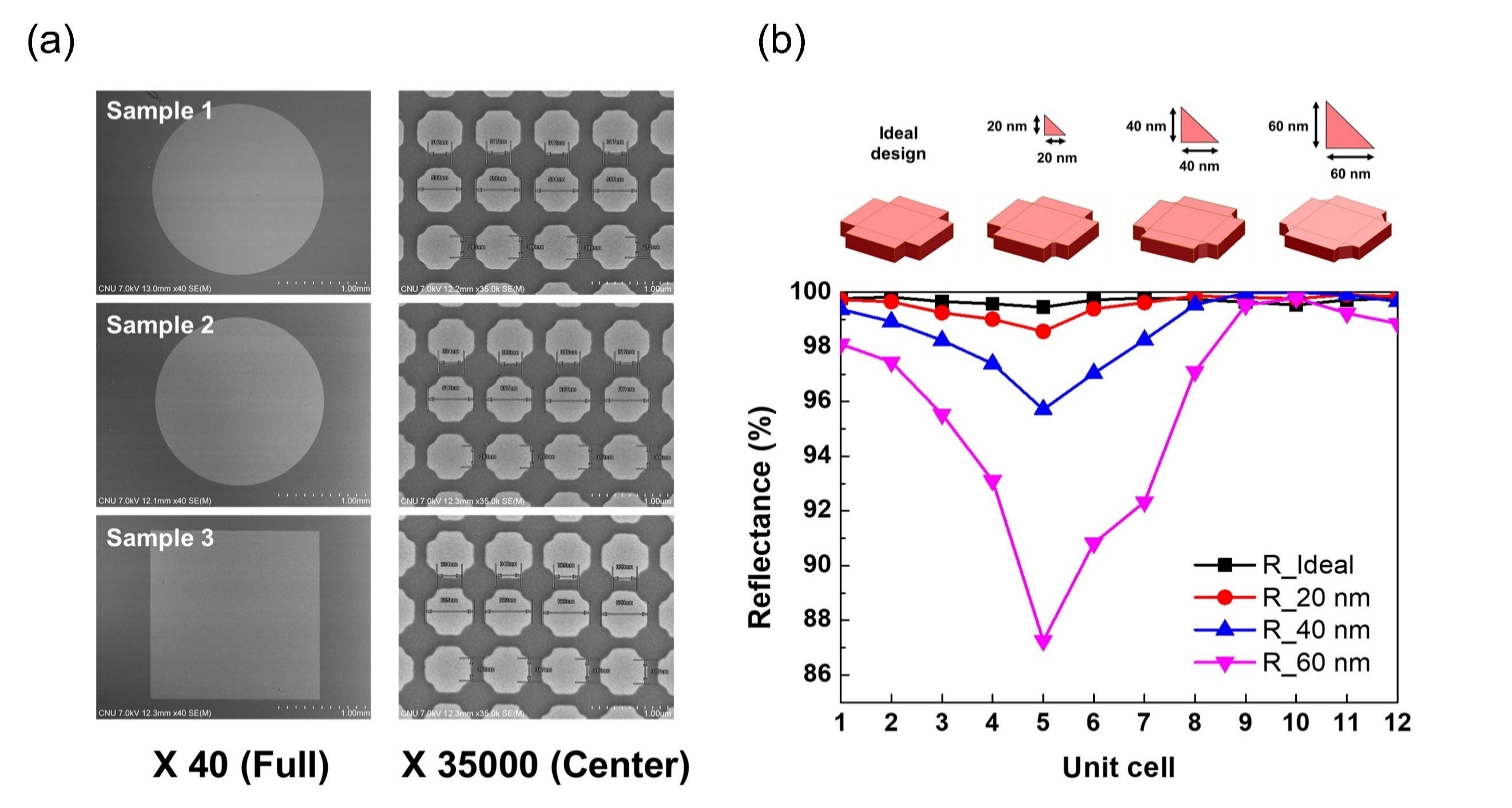


Figure S4: SEM images of the fabricated samples and reflectance calculated based on the size of the blunt corners. (a) SEM images of the three metasurfaces used for experimental measurements. The left column shows the full images, while the right column presents magnified views of the central region of the samples at 35,000x magnification. (b) Reflectance values corresponding to the size of the blunt corners for each of the 12 unit cells used in the metalens design.

**S5.** **Design of the vertical cavity with the metasurface proposed in this study and its simulation results**

We anticipate that the metasurface proposed in this study can be applied to vertical cavity devices capable of output wavefront modulation. To support this claim more convincingly, we present a schematic design and FDTD simulation results for a vertical cavity structure in which the meta-atom proposed in this study is applied as the upper reflector. First, we provide the simulation results related to the cavity characteristics resulting from the reflective effect of the high-reflectivity meta-atom proposed in this study. Figure S5(a) shows a comparison of the transmittance with and without the meta-atom. In both cases, the bottom structure is the same, consisting of DBRs made of three pairs of a-Si:H/SiO_2_ on an SiO_2_ substrate, followed by an a-Si:H cavity layer, and then a single pair of a-Si:H/SiO_2_ layers. The single pair of a-Si:H/SiO_2_ layers was chosen to slightly support field confinement to the cavity layer. On top of this, a 1280 nm-thick SiO_2_ spacer was selected, optimized to achieve a resonance wavelength of 980 nm. As a result, the right graph of Fig. S5(a) shows that the structure with the meta-atom has a significantly higher Q-factor compared to the one without. Additionally, to demonstrate that not only the cavity characteristics but also output wavefront modulation can be achieved when combining the metalens with the bottom structure, we present simulation results for a meta-cavity with a high-reflectivity metalens (53.9 µm in diameter) placed on top, as shown in Fig. S5(b). The left image shows the schematic of the proposed meta-cavity with the metalens, and the right image depicts the electromagnetic field profile for the integrated structure. The simulation results confirm that the output light is well-focused by the metalens.


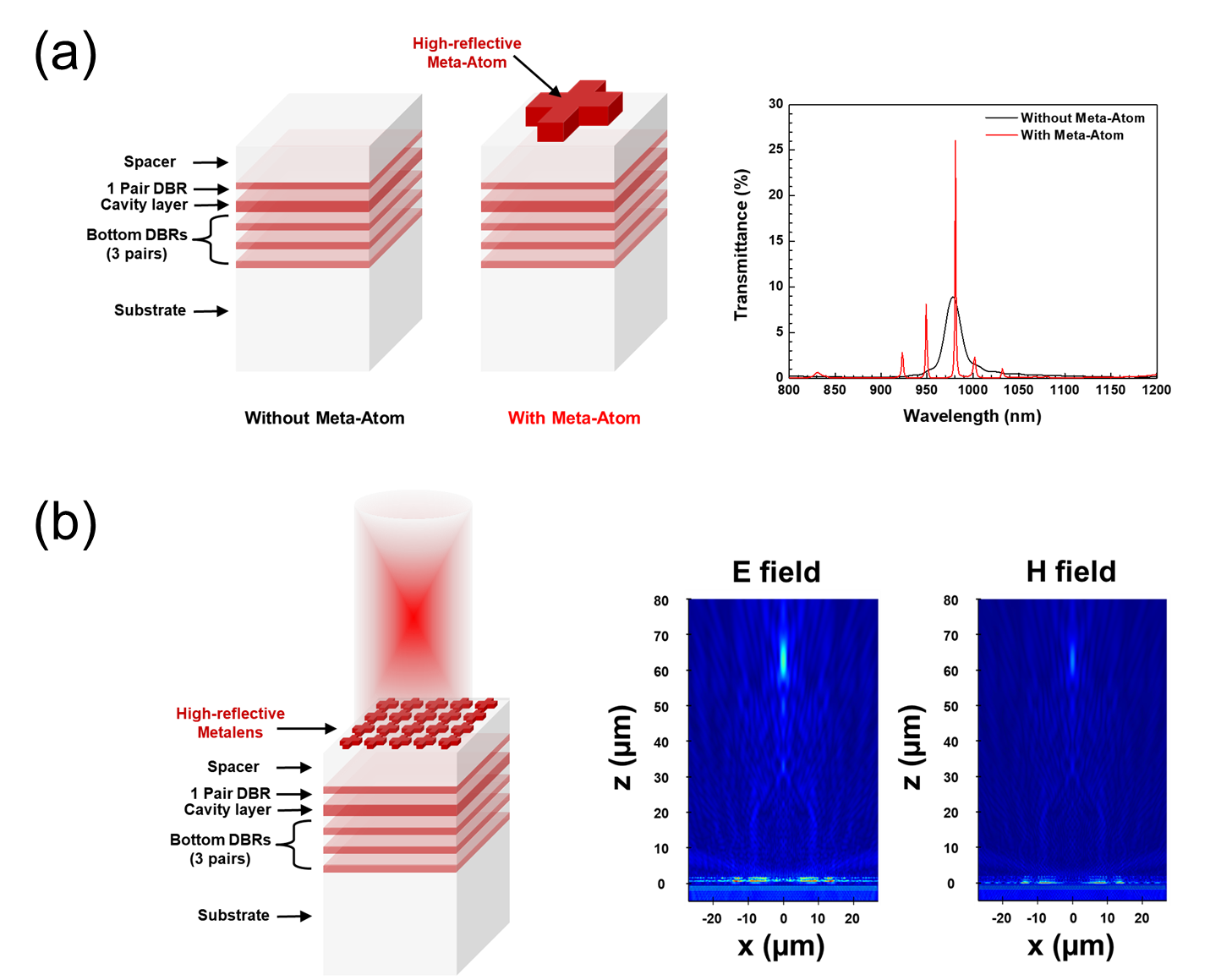


**Figure S5**: Design and simulation results of the proposed metasurface-based vertical cavity. (a) Schematic and simulation results of the vertical cavity structure with and without the upper meta-atom. The black line corresponds to the transmission spectrum without the meta-atom, while the red line corresponds to the transmission spectrum with the meta-atom. (b) Schematic of the meta-cavity with the proposed high-reflective metalens placed on top (left), and simulation results of the electromagnetic field profile for the integrated structure (right). The field profile is displayed in absolute values.
